# Transcranial Doppler pulsatility index is a poor predictor of hydrocephalus in patients with aneurysmal subarachnoid haemorrhage

**DOI:** 10.1186/cc10899

**Published:** 2012-03-20

**Authors:** MH Kiel, AW Oldenbeuving, M Sluzewski, JA Van Oers, D Ramnarain

**Affiliations:** 1 St Elisabeth Hospital, Tilburg, the Netherlands

## Introduction

Hydrocephalus is a common complication of aneurysmal subarachnoid haemorrhage (aSAH). The increase in intracranial pressure is associated with increased mortality and morbidity. Early recognition and intervention in these patients is essential in order to achieve favourable outcome. In the literature the value of noninvasive measurement of transcranial Doppler (TCD)-derived pulsatility index (PI) in predicting increased intracranial pressure remains questionable. The aim of this study was to examine the value of PI in predicting hydrocephalus in patients with aSAH.

## Methods

In a retrospective cohort study from January 2010 to June 2011, 61 patients with aSAH were diagnosed with hydrocephalus on CT scan during treatment in our ICU. On 93 occasions of TCD recordings of the middle cerebral artery, PI was calculated on the same day.

## Results

**Figure 1:**
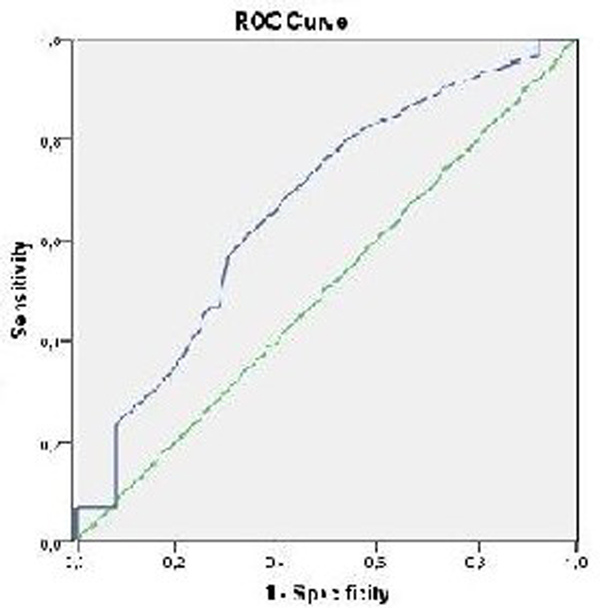
**Receiver operating characteristic curve**.

See Table 1 and Figure 1. Ninety-three CT scans could be correlated with PI on the same day of the scan. Using a cut-off value of PI >1.4, sensitivity was low (23.3%) and specificity was high (92.1%). Negative and positive predictive values were 71.6% resp. 58.3%. The receiver operating characteristic curve showed an area under the curve of 0.67. The likelihood ratio for a negative (LR-) resp. positive (LR+) test was 0.83 resp. 2.94. Pretest probability of 32% increased to 57% post-test probability with PI >1.4 and decreased to 28% with PI ≤1.4.

**Table 1:** The 2 × 2 table for PI 1.4

Index test	Hydrocephalus +	Hydrocephalus -	Total
PI >1.4	7	5	12
PI ≤1.4	23	58	81
Total	30	63	93

## Conclusion

PI with a cut-off value of 1.4 has a poor sensitivity and a high specificity. PI has limited value in ruling in and out hydrocephalus in aSAH patients due to a low LR+ and LR-.

